# Survival Outcomes in Breast Cancer Patients With Metastatic Bone Disease

**DOI:** 10.1002/wjs.70147

**Published:** 2025-10-28

**Authors:** Gerard J. Hill, Aisling Hegarty, Gavin P. Dowling, Sandra Hembrecht, Gordon R. Daly, Trudi Roche, Eithne Downey, Michael Allen, Colm Power, Nuala Healy, Leonie S. Young, Arnold D. K. Hill

**Affiliations:** ^1^ Department of Surgery RCSI University of Medicine and Health Sciences Dublin Ireland; ^2^ Beaumont RCSI Cancer Centre Beaumont Hospital Dublin Ireland

**Keywords:** bone metastasis, breast cancer, survival

## Abstract

**Objective:**

To evaluate the survival outcomes of breast cancer patients with metastatic bone disease and to assess whether these patients exhibit different prognoses compared to those with more extensive metastatic involvement.

**Background:**

Systemic therapy including endocrine therapy, chemotherapy and targeted agents remains the cornerstone of treatment for patients with stage IV breast cancer, particularly those with bone metastases. Palliative radiotherapy also plays a key role in bone metastases, especially for symptom control and managing skeletal and complications. Although these modalities have significantly improved outcomes, the prognostic variation among patients with bone‐only disease—ranging from solitary to widespread metastases—warrants further investigation. This study aims to evaluate survival outcomes among breast cancer patients with varying patterns of bone metastases.

**Methods:**

This retrospective cohort study analyzed data from 4000 breast cancer patients treated between 2006 and 2024. Patients were evaluated for bone metastases, which were confirmed through imaging reviewed via the Picture Archiving and Communication System (PACS). Patients with confirmed bone metastases were categorized into solitary, oligometastatic (2–5 sites), or multiple metastases groups. Data on demographics, tumor characteristics, treatment regimens, surgery types, and survival outcomes were collected. Survival analyses were conducted using Kaplan–Meier curves and compared using log‐rank tests.

**Results:**

One hundred and eighty‐seven patients with metastatic bone disease were identified. 21 patients had solitary bone metastases only with a mean survival of 14.6 years. Additionally, 30 patients had oligometastatic bone disease only with a mean survival of 7 years. Another 31 patients had multiple bone only metastases, with also a mean survival of 7 years. Finally, 105 of the 187 patients had other metastases alongside bone metastases in other organs including the lung, liver, and brain with a mean survival of 6.3 years.

**Conclusion:**

These findings suggest that patients with bone‐only metastases, particularly those with solitary lesions, exhibit significantly longer survival. Although systemic therapy remains the standard these findings suggest that patients with bone‐only metastases, particularly those with solitary lesions, exhibit significantly longer survival. Although systemic therapy remains the standard, these findings highlight the need for further research into whether selected patients may benefit from integrating local treatment approaches, including surgery, into their management.

## Graphical abstract

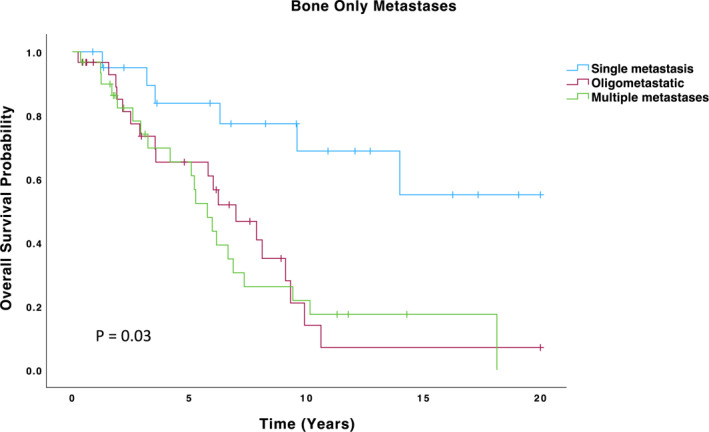

## 1 Introduction

Breast cancer is the most common malignancy among women worldwide, and a significant proportion of patients present with, or develop, metastatic disease, often involving the bone [1]. Metastasis occurs when cancer cells spread from the primary tumor to distant sites in the body, with the skeleton being one of the most common destinations [2]. Bone metastasis occurs in up to 70% of patients with advanced breast cancer, leading to significant morbidity due to pain, fractures, and other skeletal‐related events [3].

For patients diagnosed with stage IV breast cancer with bone metastases, the standard treatment approach has traditionally focused on systemic therapy, including chemotherapy, hormonal therapy, and targeted treatments [4]. These systemic therapies aim to control disease progression, alleviate symptoms, and prolong survival. Radiotherapy also plays an important role in managing bony metastases, particularly for pain relief, with advancements such as stereotactic ablative body radiotherapy (SABR) improving treatment efficacy [5, 6].

Bone metastases in breast cancer can present as solitary, oligometastatic (2–5 sites), or multiple metastases. Solitary bone metastases refer to the presence of a single metastatic lesion, which may indicate an early or limited metastatic burden [7]. Oligometastatic bone disease is characterized by a limited number of metastatic lesions, often defined as 2–5 sites [8]. Multiple metastatic bone disease involves widespread skeletal involvement with numerous metastatic sites. However, survival outcomes across the spectrum of bone metastases in breast cancer patients remain poorly characterized.

This study aims to evaluate survival outcomes in breast cancer patients with bone disease. Specifically, we sought to assess whether patients with bone‐only metastases particularly those with solitary or oligometastatic involvement exhibit differential survival outcomes compared to those with more widespread disease. By analyzing an 18‐year institutional dataset of over 4000 patients, we seek to characterize the clinical trajectory of these subgroups and explore potential factors associated with prolonged survival. Our findings aim to highlight survival variation within bone metastatic subgroups which may help generate hypotheses for future prospective studies.

## 2 Methods

This retrospective cohort study was conducted using data from a prospectively maintained database of breast cancer patients treated at a single institution from January 2006 to September 2024. The database included over 4000 patients who were diagnosed with breast cancer during this period. Our study evaluated the presentation, management, and survival outcomes of patients who developed metastatic bone disease secondary to breast cancer.

### 2.1 Patient Identification and Data Collection

Clinical data for all patients were collected from the clinical trial “Breast Cancer Proteomics and Molecular Heterogeneity” (ClinicalTrials.gov identifier: NCT01840293). Informed written consent was obtained from all patients prior to the collection of any clinical data. The study received approval from the Beaumont Hospital Research Ethics Committee (REC), with REC reference number 13/09. All patients included in the study had provided consent for future research use of their data. This research was conducted as a single‐center, retrospective observational cohort study of a prospectively maintained database. All patients within the database were reviewed to determine if they had developed bone metastases during their disease course. Patients were included if bone metastases were present at initial staging or developed during follow‐up. Detailed data was gathered from patient medical records, including demographics, tumor and pathological characteristics, treatment regimens, surgery type and survival outcomes. The presence of bone metastases was initially identified through clinical records and confirmed through imaging studies. The Picture Archiving and Communication System (PACS) was utilized to review and verify imaging data using X‐rays, CT scans, MRI scans, and bone scans. The presence of histological confirmation of bone metastases was also recorded.

The 2010 American Society of Clinical Oncology/College of American Pathologists (ASCO/CAP) histopathological consensus guidelines were utilized to record estrogen (ER) and progesterone (PR) receptor status [9, 10]. HER2 status was initially determined using immunohistochemistry (IHC) and patients with a 2+ (equivocal) score underwent additional fluorescence in situ hybridization (FISH) for confirmation of HER2 status.

### 2.2 Inclusion and Exclusion Criteria

All patients included in this study were initially seen through the symptomatic breast clinic at our institution between January 2006 and September 2024. These patients were diagnosed with breast cancer and subsequently developed bone metastases, either identified at initial staging or during follow‐up.

Patients were included if they:

- Were 18 years or older.
- Had a histologically confirmed diagnosis of breast cancer.
- Were diagnosed with bone metastases, either at initial staging or during clinical follow‐up.

Patients were excluded if they:

- Were under 18 years at diagnosis.
- Were pregnant at the time of diagnosis.
- Had bone metastases with an unknown or unconfirmed primary tumor.

### 2.3 Statistical Analysis

Clinicopathological characteristics, IHC, treatment details, and clinical outcomes were analyzed using descriptive statistics. Fisher's exact (¶) and Chi‐squared (*χ* ^2^) were applied where appropriate. All tests of significance were 2‐tailed, with a threshold of *p* < 0.05 considered statistically significant. Kaplan–Meier and Log‐rank (Mantel‐Cox) analyses were conducted to compare survival differences across the various cohorts. Data analysis was performed using the Statistical Package for Social Sciences (SPSS) Version 26 (International Business Machines Corporation, Armonk, New York).

## 3 Results

One‐hundred‐and‐eighty‐seven breast cancer patients with confirmed bone metastases were identified from our cohort of over 4000 breast cancer patients treated between 2006 and 2024. The baseline characteristics of these patients, including age at diagnosis, hormone receptor status, tumor size, lymph node involvement, type of surgery performed and the number of patients who underwent bone biopsies are summarized in Table 1. The number of patients diagnosed with bone metastases at the time of primary breast cancer diagnosis (synchronous) and those diagnosed during follow‐up (metachronous) are also included in Table 1. The majority underwent surgery initially (110 mastectomy), 34 breast conserving surgery (BCS) however 43 patients presented with de novo metastatic disease. The diagnosis of bone metastases was confirmed through imaging studies such as CT scan, bone scan (skeletal scintigraphy), MRI or X‐Ray, with the specific imaging modality and location of bone involvement presented in Table 2. The majority of bone metastases were located in the thoracic and lumbar spine.

**TABLE 1:** Characteristics of the 187 breast cancer patients diagnosed with bone metastases.

Trait		All (*n* = 187)	Solitary (*n* = 50)	Oligometastatic (*n* = 62)	Multiple (*n* = 75)	Solitary (bone only) (*n* = 21)	Oligometastatic (bone only) (*n* = 30)	Multiple (bone only) (*n* = 31)
Age at diagnosis	Mean ± SD (range); median	52.2 ± 14 (21–87); 49	52.2 ± 12.42 (32–78); 50	50.5 ± 16 (21–87); 46	53.5 ± 13.1 (28–82); 50	53 ± 13 (34–78); 51	53 ± 16.8 (21–87); 47	56.9 ± 13.3 (29–82); 59
ER+	*N* (%)	149 (79.7%)	37 (74%)	47 (75.8%)	65 (86.6%)	17 (81%)	21 (70%)	26 (83.9%)
ER‐ER (U)		31 (16.6%) 7 (3.7%)	10 (20%) 3 (6%)	13 (21%) 2 (3.2%)	8 (10.6%) 2 (2.6%)	1 (4.8%) 3 (14.3%)	5 (23.8%) 4 (13.3%)	4 (12.9%) 1 (3.2%)
PR+	*N* (%)	112 (59.9%)	24 (48%)	42 (67.7%)	46 (61.3%)	12 (57.1%)	17 (56.6%)	23 (74.2%)
PR‐PR (U)		66 (35.3%) 9 (4.8%)	21 (42%) 5 (10%)	18 (29%) 2 (3.2%)	27 (36%) 2 (2.6%)	4 (19%) 5 (23.8%)	9 (30%) 4 (13.3%)	8 (25.8%) 0
HER2+	*N* (%)	42 (22.5%)	17 (34%)	11 (17.7%)	14 (18.6%)	4 (19%)	5 (16.6%)	6 (19.4%)
HER2‐HER2 (U)		133 (71.1%) 12 (6.4%)	29 (58%) 4 (8%)	47 (75.8%) 4 (6.5%)	57 (76%) 4 (5.3%)	14 (66.6%) 3 (14.3%)	22 (73.3%) 3 (10%)	25 (80.6%) 0
pT1		26 (14%)	9 (18%)	10 (16.1%)	7 (9.3%)	4 (19%)	6 (20%)	1 (3.2%)
pT2		89 (47.6%)	8 (16%)	15 (24%)	12 (16%)	3 (14.3%)	9 (30%)	6 (19.4%)
pT3		62 (33.2%)	15 (30%)	18 (29%)	29 (38.6%)	6 (28.6%)	7 (23.3%)	11 (35.5%)
pN0	*N* (%)	30 (16%)	10 (20%)	7 (11.2%)	13 (17.3%)	6 (28.6%)	6 (20%)	4 (12.9%)
pN1‐3		162 (86.6%)	38 (76%)	52 (83.9%)	58 (77.3%)	14 (66.6%)	22 (73.3%)	25 (80.6%)
BCS	*N* (%)	34 (18.2%)	13 (26%)	11 (17.7%)	10 (13.3%)	5 (23.8%)	6 (20%)	4 (12.9%)
Mastectomy		110 (58.8%)	28 (56%)	36 (58%)	46 (61.3%)	13 (61.9%)	17 (56.6%)	16 (51.6%)
Synchronous bone mets		55 (29.4%)	15 (30%)	17 (27.4%)	23 (30.6%)	6 (28.6%)	9 (30%)	13 (41.9%)
Metachronous bone mets		132 (70.6%)	35 (70%)	45 (72.6%)	52 (69.3%)	15 (71.4%)	21 (70%)	18 (58.1%)
Bone biopsy		94 (50.3%)	26 (52%)	29 (46.8%)	39 (52%)	18 (85.7%)	20 (66.6%)	22 (71%)

**TABLE 2:** Radiological data of type of imaging and location of Bone metastases diagnoses.

Site	CT	Bone scan	MRI	X‐ray
Cervical spine	25 (7.3%)	21 (6.1%)	10 (8.2%)	2 (6.5%)
Clavicle	2 (0.5%)	1 (0.29%)	0	0
Femur	20 (5.9%)	27 (7.8%)	7 (5.7%)	3 (9.7%)
Humerus	9 (2.6%)	15 (4.3%)	4 (3.3%)	2 (6.5%)
Lumbar spine	61 (18%)	52 (15%)	26 (21.3%)	5 (16.1%)
Pelvis	41 (12.1%)	40 (11.6%)	7 (5.7%)	6 (19.4%)
Ribs	38 (11.2%)	54 (15.7%)	8 (6.6%)	3 (9.7%)
Sacrum	29 (8.6%)	21 (6.1%)	13 (10.7%)	3 (9.7%)
Scapula	10 (3%)	8 (2.3%)	1 (0.8%)	0
Skull	10 (3%)	17 (5%)	4 (3.2%)	1 (3.2%)
Sternum	28 (8.3%)	36 (10.4%)	6 (4.9%)	0
Thoracic spine	65 (19.2%)	53 (15.4%)	25 (20.5%)	6 (19.4%)
Tibia	0	0	1 (0.8%)	0
Total	338	345	112	31

Among the 187 patients, 149 were estrogen receptor (ER)‐positive, based on immunohistochemical analysis of the primary tumor, highlighting the prevalence of ER‐positive disease in breast cancer with bone metastasis. Of the 21 patients with solitary bone‐only metastases, 17 (81%) were ER‐positive, and of the 30 patients with oligometastatic bone‐only disease, 21 (70%) were ER‐positive. In terms of HER2 status, 42 out of the 187 patients (22%) were HER2‐positive, as shown in Table 1. Of the 21 patients with solitary bone‐only metastases, 4 (19%) were HER2‐positive, and 9 (30%) of the 30 patients with oligometastatic bone‐only disease were HER2‐positive.

**FIGURE 1:**
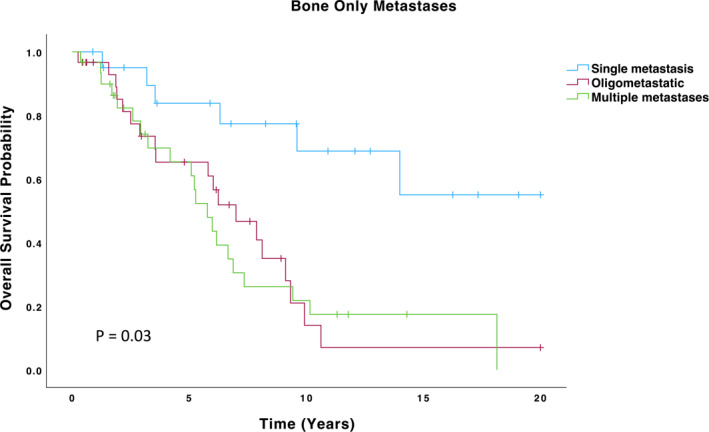
Overall survival in patients with bone only metastases.

Among these 187 patients, 21 were found to have solitary bone‐only metastases, with a mean survival of 14.6 years, a particularly favorable prognosis (Figure 1, Table 3). Six of these patients were diagnosed with solitary metastases at the time of their initial breast cancer diagnosis. Notably, two of these patients underwent resection of the primary breast tumor, which was associated with a particularly favorable prognosis, as both patients underwent surgery in 2012 and remain alive 12 years later.

**TABLE 3:** Overall survival times of bone‐only metastasis groups.

Group	Mean estimate	Std. Error	95% CI (lower)	95% CI (upper)	Median (95% CI)
Single	14.60	1.76	11.16	18.04	—
Oligo	7.03	1.05	4.98	9.08	6.99 (4.60–9.38)
Multiple	7.17	1.16	4.89	9.45	5.77 (4.60–6.94)
Overall	9.19	0.87	7.48	10.89	6.99 (5.09–8.89)

A further 30 patients were identified with oligometastatic bone‐only disease, defined as metastases involving 2 to 5 sites. This group had a mean survival of 7 years (Figure 1, Table 3). Additionally, 31 patients had multiple bone‐only metastases, with a mean survival of 7 years, similar to the oligometastatic group (Figure 1, Table 3). These findings suggest that in patients with bone‐only disease, the number of metastatic sites (whether oligometastatic or multiple) does not significantly impact overall survival, as both groups displayed comparable outcomes. The treatments received by patients including systemic chemotherapy, endocrine therapy, targeted agents, bone‐modifying agents, and radiotherapy to the primary tumor are summarized in Table 4.

**TABLE 4:** Treatment modalities by each Metastatic Subgroup.

Treatment	All (*n* = 187)	Solitary (*n* = 50)	Oligo‐metastatic (*n* = 62)	Multiple (*n* = 75)	Solitary (bone only) (*n* = 21)	Oligo‐metastatic (bone only) (*n* = 30)	Multiple (bone only) (*n* = 31)
Alkylating agent‐based	48	12	19	17	5	7	7
Antimetabolite‐based	20	6	5	9	2	2	1
Anthracycline‐based	13	6	3	4	4	1	3
Taxane‐based	16	4	6	5	0	2	1
Bone‐targeted therapy	10	1	4	5	0	3	4
Endocrine therapy	13	3	4	6	1	2	4
Platinum‐based	5	2	1	2	1	0	2
Targeted therapy (CDK/PI3K/HER2)	4	0	2	2	0	1	0
Untreated (De Novo/declined/unfit)	58	16	18	24	8	12	9
Radiotherapy to primary	131	37	41	53	15	19	17

**FIGURE 2:**
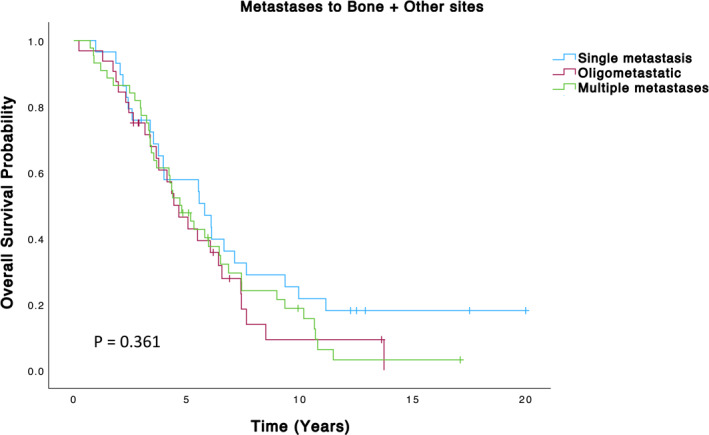
Overall survival in patients with bone metastases and metastases at other sites.

The remaining 105 patients had metastases to other organs in addition to the bone, including the lung, liver, and brain. This group was further subdivided into solitary (*n* = 29), oligometastatic (*n* = 32), and multiple metastases (*n* = 44) (Figure 2, Table 5). Among the 29 patients with solitary metastases to other organs, the mean survival was 7.7 years, which was significantly lower than the survival of patients with solitary bone‐only metastases. The oligometastatic group had a mean survival of 5.4 years, whereas the group with multiple metastases had a mean survival of 5.8 years. Overall, this subgroup of patients with metastases to other organs had a mean survival of 6.3 years and median survival of 5 years, which is notably lower than that of patients with bone‐only metastases (Table 5). Median survival could not be estimated for the “Solitary bone‐only metastases” group due to a high proportion of censored cases and survival probability remaining above 50% throughout the observed period.

**TABLE 5:** Survival of Bone + other metastases groups.

Group	Mean estimate	Std. Error	95% CI (lower)	95% CI (upper)	Median (95% CI)
Single	7.70	1.19	5.38	10.03	5.79 (3.12–8.46)
Oligo	5.39	0.66	4.09	6.69	4.43 (3.26–5.60)
Multiple	5.77	0.58	4.64	6.89	4.69 (3.59–5.81)
Overall	6.32	0.51	5.33	7.32	5.05 (3.93–6.18)

## 4 Discussion

In the staging of breast cancer, metastasis to the bone is classified as stage IV disease, historically associated with a poor prognosis and reduced treatment options [11]. As a result, systemic therapies have traditionally formed the cornerstone of management, with local treatment such as surgery rarely recommended in this setting [12]. This approach is grounded in the belief that advanced‐stage disease should prioritize systemic control and symptom management over local intervention.

However, advancements in medical treatments, particularly the introduction of bisphosphonates, denosumab, and more effective systemic therapies such as targeted agents and CDK4/6 inhibitors as well as targeted radiotherapy approaches, have significantly improved the prognosis for patients with bone‐only metastases [12]. These therapies not only reduce skeletal‐related events (such as fractures and spinal cord compression) but also contribute to prolonged survival in many cases. As a result, there has been a shift in how stage IV breast cancer with limited bone metastases is viewed, prompting reconsideration of the role of surgery in selected cases.

In this study, we have reviewed our institutional experience of 187 breast cancer patients who developed bone metastases. Our findings suggest that, for some patients particularly those with solitary bone only disease, may have a more favorable prognosis than historically perceived. This observation raises the possibility that selected patients could benefit from more individualized or intensified treatment approaches. Although a small number of patients who underwent surgery appeared to have good outcomes, the sample size is too limited to draw firm conclusions regarding the role of surgical intervention. Notably, two large randomized controlled trials found no survival advantage from surgical removal of the primary tumor in patients with synchronous metastatic breast cancer, highlighting the need for cautious patient selection when considering local interventions [13, 14]. It should be emphasized that any consideration of surgery must be viewed in the context of systemic therapies, which remain the foundation of treatment. The effectiveness of hormonal, HER2‐targeted, and chemotherapeutic agents often shapes whether local interventions are pursued. As such, although surgery may represent a potential option for carefully selected individuals, further investigation is required to clarify its impact in this context.

Our findings highlight a significant difference in survival outcomes between patients with solitary bone metastases and those with both bone and other organ involvement. Patients with a single bone‐only metastasis exhibited a mean survival of 14.6 years, whereas those with solitary bone metastasis alongside metastases to other organs had a mean survival of 6.3 years. This difference suggests that patients with solitary bone metastasis may have a more favorable prognosis. Our findings reinforce the need for continued investigation into the optimal management of patients with limited metastatic disease, including whether selected individuals may benefit from more aggressive or localized approaches.

Similarly, for patients with oligometastatic bone disease, emerging treatments such as stereotactic body radiation therapy (SBRT) has shown promise, although longer follow up is needed to determine the extent of this [15, 16]. Metastasectomy, the surgical resection of metastatic lesions, is a well‐established component of treatment in several malignancies, including colorectal cancer with liver metastases and renal cell carcinoma with pulmonary metastases. In selected patients, resection of limited metastatic disease has been associated with improved survival outcomes [17]. Its role as a method of disease control in breast cancer patients with bony metastases merits further research [13, 15, 17, 18, 19].

A notable feature of our cohort was the predominance of ER‐positive disease among patients with bone metastases. Of the 187 patients identified, 149 (79.7%) were ER‐positive and 42 (22.5%) were HER2‐positive. This biomarker distribution aligns with the well‐established tendency of ER+ and HER2+ breast cancers to metastasize to bone [20]. However, given the small size of biomarker defined subgroups (e.g., only 4 HER2+ patients in the solitary bone metastasis cohort), we were unable to perform a reliable survival analysis stratified by receptor status. As such, we have refrained from making direct conclusions about the prognostic implications of molecular subtype within our dataset.

Nonetheless, the high proportion of ER+ patients in this cohort is clinically significant. Hormonal therapies, including selective estrogen receptor modulators (SERMs) such as tamoxifen, aromatase inhibitors such as letrozole and anastrozole, and selective estrogen receptor degraders (SERDs) such as fulvestrant, have proven to be highly effective in treating ER‐positive breast cancer [21]. The development of CDK4/6 inhibitors, when combined with hormonal therapies, has further enhanced outcomes for these patients, offering durable responses and delaying disease progression [22]. In the context of our study, the high prevalence of ER positivity in patients with bone metastasis emphasizes the importance of leveraging these therapies to maximize survival outcomes.

Similarly, HER2+ disease has benefitted substantially from targeted therapies such as trastuzumab and pertuzumab, with novel agents such as trastuzumab‐deruxtecan (T‐DXd) also showing promising results [23]. Antihormonal and anti‐HER2 therapies have revolutionized the treatment landscape for breast cancer, especially for patients with ER‐positive and HER2‐positive disease [21, 24]. It is possible that, in the decades ahead, systemic therapies may become so advanced that breast cancer could be treated entirely through these medical approaches, reducing the need for surgical intervention to the primary tumor [25].

However, at present, these therapies—though highly effective—are not 100% curative. Both antihormonal and anti‐HER2 therapies, although capable of significantly delaying disease progression and improving survival, still have limitations, with some patients experiencing resistance or incomplete responses [25, 26]. For this reason, we believe that surgery to the primary tumor should still be considered for certain subgroups of patients in select cases, particularly those with a favorable prognosis.

This study benefits from a large, prospectively maintained single‐institution database spanning over 18 years, providing a robust longitudinal real‐world perspective on metastatic breast cancer patients with bone involvement. It offers important insight into survival outcomes in this subgroup, which is often underrepresented in clinical trials. However, several limitations must be acknowledged. Firstly, the retrospective design introduces potential biases, including selection bias. Secondly, the relatively limited numbers of patients within key subgroups, in particular those with solitary bone metastases, limit the statistical power to perform multivariable analyses or draw definitive conclusions regarding the impact of surgery or systemic therapies. Thirdly, treatment approaches evolved significantly over the study period, potentially confounding survival comparisons. Future prospective studies are warranted to validate these findings and explore optimal treatment strategies for patients with limited bone metastases.

In conclusion, patients with bone‐only metastatic breast cancer, particularly those with solitary lesions may experience significantly prolonged survival, reflecting a more favorable disease biology. Although systemic therapy remains the cornerstone of treatment, these findings support further investigation into the role of local therapies, including surgery and SBRT, for selected patients. Larger prospective studies are needed to better define treatment strategies in this underrepresented subgroup and determine whether integrating local and systemic approaches can optimize outcomes [13, 14].

## Author Contributions

**Gerard J. Hill:** conceptualization, investigation, data curation, writing – original draft, writing – review and editing, visualization. **Aisling Hegarty:** conceptualization, methodology, software, validation, formal analysis, investigation, resources, data curation, writing – original draft, writing – review and editing, visualization, project administration. **Gavin P. Dowling:** conceptualization, methodology, software, validation, formal analysis, investigation, resources, data curation, writing – original draft, writing – review and editing, visualization, project administration. **Sandra Hembrecht:** data curation, writing – original draft, writing – review and editing. **Gordon R. Daly:** methodology, formal analysis, investigation, data curation, writing – original draft, writing – review and editing, visualization. **Trudi Roche:** validation, investigation, resources, data curation, writing – original draft, writing – review and editing, project administration. **Eithne Downey:** resources, data curation, project administration. **Michael Allen:** investigation, writing – review and editing. **Colm Power:** investigation, resources, writing – review and editing, project administration. **Nuala Healy:** methodology, investigation, resources, writing – review and editing, supervision, project administration. **Leonie S. Young:** investigation, resources, data curation, writing – review and editing, supervision, project administration. **Arnold D. K. Hill:** conceptualization, methodology, software, validation, formal analysis, investigation, resources, data curation, writing – original draft, writing – review and editing, visualization, supervision, project administration.

## Funding

The authors have nothing to report.

## Conflicts of Interest

The authors declare no conflicts of interest.

## Data Availability

The data that support the findings of this study are available on request from the corresponding author. The data are not publicly available due to privacy or ethical restrictions.
